# Age at Surgery and Outcomes of Undescended Testes at King Salman Armed Forces Hospital, Tabuk, Saudi Arabia

**DOI:** 10.7759/cureus.6413

**Published:** 2019-12-18

**Authors:** Mohammad S Mohammad Alnoaiji, Tahani N Alrashidi, Asmaa S Ghmaird, Sarah S Alsalem, Malak S Alanazi, Alanuad I Albazei, Maryam O Alenzi, Mastorah A Aljuhani, Rana S Alotaibi, Sara A Alanazi, Aeshah M Althomali, Ahad M Almohammadi, Eid H Alshahrani

**Affiliations:** 1 Paediatric Surgery, King Salman Armed Forces Hospitals, Tabouk, SAU; 2 Pediatric Surgery, University of Tabuk, Tabuk, SAU; 3 Pediatrics, University of Tabuk, Tabuk, SAU; 4 Otolaryngology Head and Neck Surgery, University of Tabuk, Tabuk, SAU; 5 Pediatric Surgery, University of Bisha, Bisha, SAU

**Keywords:** cryptorchidism, orchidopexy, undescended testis, age at surgery, outcomes

## Abstract

Objective

The aim of this study was to investigate the age at diagnosis and surgery of undescended testes and patients’ outcomes.

Methods

This is a retrospective study that reviewed the files of patients who underwent orchidopexy at the King Salman Armed Forces Hospital (KSAFH), Tabuk, Saudi Arabia (SA), between January 1, 2015, and March 30, 2019. All children from birth until 13 years old who were admitted within the specified time frame and underwent orchidopexy were included in this study. The gathered data were analyzed through the Statistical Package for Social Sciences software (SPSS, version 23; SPSS Inc., Chicago, IL, USA).

Results

A total of 175 patients were included in this study. The rate of orchidopexy at our institution was 12.2%. The median ages at diagnosis and surgery were 12 and 24 months, respectively. The median duration between diagnosis and surgery was eight months. The most common site of undescended testis was inguinal (80.6%). Bilateral undescended testes were recorded in 24.6% of cases, and 25.7% of cases were impalpable. The size of the undescended testis was average in half the cases, small in 44.6% and atrophic in 6.4% of cases. Postoperative complications were reported in 4.0% of cases. Cox regression analysis revealed that the age at diagnosis was a significant risk factor affecting the time of surgery.

Conclusion

The findings of this study revealed that most cases of undescended testes in Tabuk were operated beyond the age recommended by international guidelines. The age at diagnosis seems to significantly affect the time of surgery.

## Introduction

Undescended testis (UDT) is a condition in which the testes do not descend to a normal position in the scrotum. It can be unilateral or bilateral. UDT is considered one of the major genital anomalies in young males [1]. Due to the continuous spontaneous descent of the testes, the prevalence by the age of three months decreases to 1% to 2% [2]. After 4 to 6 months, the UDT less likely descends spontaneously. Consequently, approximately one-third of UDT cases continue to exist. This condition requires surgical intervention with orchidopexy to fix the testes within scrotum [3]. The preferred age for orchiopexy is before the completion of the first year of life. Surgery during this specified period may have optimal outcomes in fertility and protection against testicular cancer [4]. Therefore, the earlier the patient undergoes surgery, the better the outcome is expected to be. Current guidelines recommend orchidopexy at 6 to 12 months old and no later than 18 months old [5-6]. There are no previous studies on the prevalence, age at surgery and outcomes of UDT in Tabuk. An assessment of the prevalence of UDT, as well as the age at diagnosis and surgery, can help estimate adherence to current guidelines and can prompt education campaigns for physicians and parents to highlight the problem of delayed treatment of UDT. Hence, the aim of this study was to investigate the prevalence of UDT, the age of affected boys at the time of surgery and the outcomes of surgery at the King Salman Armed Forces Hospital (KSAFH), Tabuk, Saudi Arabia (SA). 

## Materials and methods

This retrospective study involved the review of the hospital files of children with UDT who underwent orchidopexy operation at KSAFH, Tabuk, SA, between January 1, 2015, and March 30, 2019. The study was approved by the Research Ethics Committee of KSAFH, Tabuk, SA. KSAFH is a secondary hospital and considered one of the major hospitals in Tabuk City. It was established in 1979, with a bed capacity 900 beds. The study protected participants’ privacy. Investigators were responsible for ensuring the security of the data. The participants’ data were not used for any purpose outside this study. Personal data (e.g., name, contact info) were not entered in the datasheet software to protect the participants' privacy. Each subject was given a unique identifier code. Sample size was all children with UDT from birth until 13 years old who were admitted within the specified time frame and who underwent orchidopexy. Inclusion criteria were patients diagnosed undescended testis and patients younger than 13 years old at the time of orchidopexy. Exclusion criteria were patients older than 13 years old at the time of orchidopexy and incomplete data in hospital medical records. Patients’ data were extracted from their medical records using their medical record number (MRN). Data were recorded into an Excel datasheet. The collected data included the following: a) age at diagnosis and surgery; b) year of surgery; c) the side of the UDT (right, left or bilateral); d) whether the testis was palpable on clinical examination; e) site of the UDT; f) type of surgical technique; and g) postoperative complications. Patients were divided into two groups according to the timing of the surgery: at/before 18 months of age (group 1) or later (group 2). Data management and analysis plan: The study variables were coded and analyzed using the Statistical Package for Social Sciences software (SPSS, version 23; SPSS Inc., Chicago, IL, USA). Categorical data were summarized as frequencies (numbers and percentages); associations between groups and categorical variables were tested using Pearson’s chi-square tests, Fisher’s exact tests, or Fisher-Freeman-Halton exact tests as appropriate. The distribution of numerical variables was tested using Shapiro-Wilk tests, and none of the variables were found to have a normal distribution. The data were then described using medians and interquartile ranges (expressed as 25th to 75th percentiles). Comparisons between the studied groups were done using Mann-Whitney U tests. Cox regression analysis was carried out to identify potential risk factors for delayed orchidopexy. Significance was adopted at *p* <0.05 for all tests.

## Results

The hospital files of pediatric patients undergoing surgery at our institution were reviewed. The total number of pediatric surgeries performed during the study period was 1436. Out of these patients, 175 underwent orchidopexy and met the inclusion and exclusion criteria of this study. Thus, the rate of orchidopexy at our institution was 12.2%.

**Figure 1:**
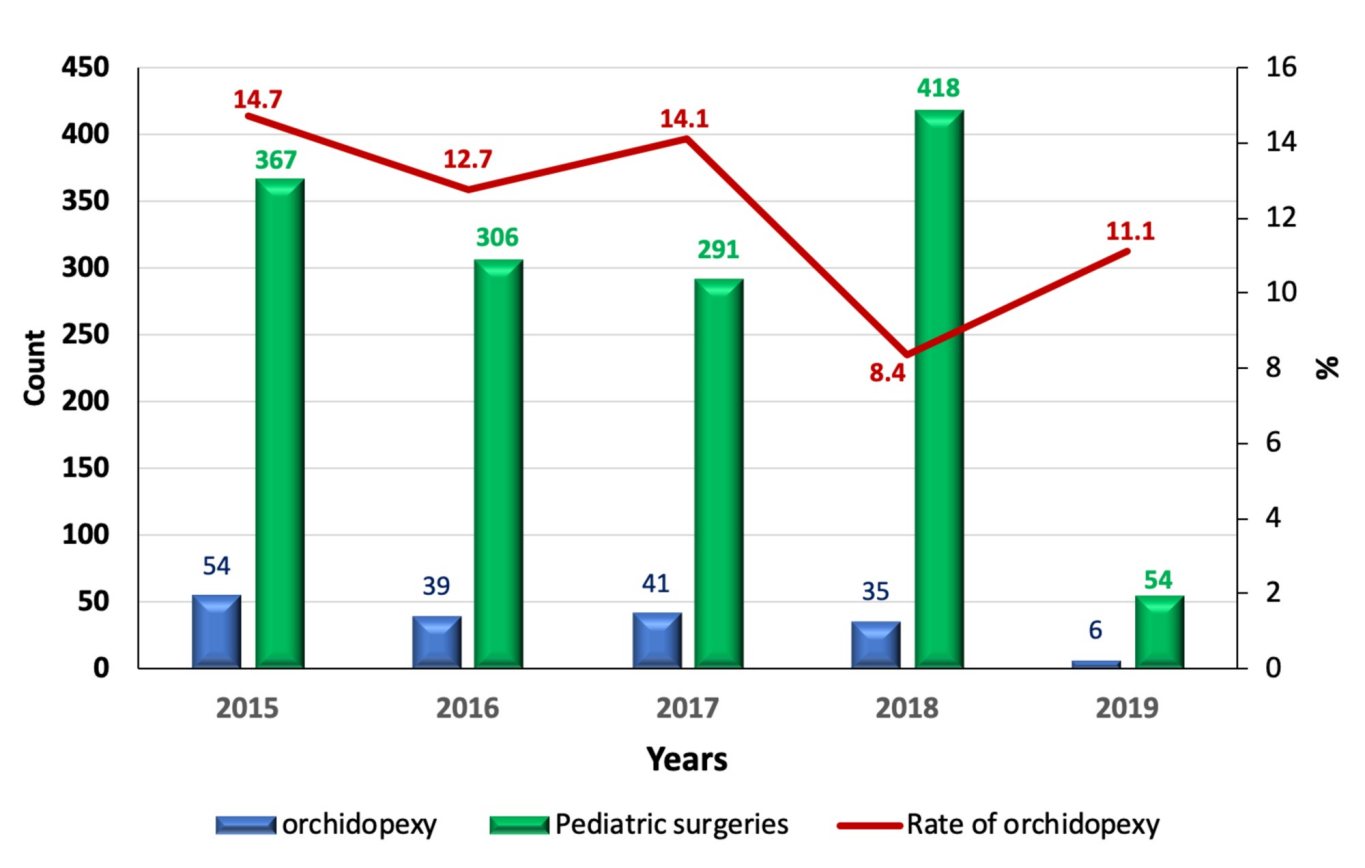
Temporal trends in the rate of orchidopexy operations at our institution during the time of the study

The temporal trend of orchidopexy operation at our institution was also analyzed. The highest number of orchidopexy operations was carried out in 2015 (n = 54), followed by 2017 (n = 41), 2016 (n = 39) and 2018 (n = 35). The study included only three months of the year 2019; therefore, only six cases were included in our study from this year. The average number of orchidopexy operations per year was 40. The rate of orchidopexies decreased in 2018 (8.4%) compared to the rate in previous years (12.7% to 14.7%; Figure 1).

Statistical analysis showed that the median age was 12 months at diagnosis and 24 months at surgery. The median duration between diagnosis and surgery was eight months. With respect to patients who underwent the two stages of the operation, the median age was 18 and 27 months at stages I and II, respectively. The median duration between the two stages was 12 months. Patients were divided into two groups according to their age at surgery. Only 66 cases out of 175 (37.7%) underwent surgery during the recommended age period (at/before 18 months old). The median age at diagnosis was significantly higher in group 2 than in group 1 (23 months versus three months, *p* < 0.001). However, there was no significant difference between the two groups with respect to the duration between diagnosis and surgery (*p* = 0.558), age at stage I (*p* = 0.057), age at stage II (*p* = 0.057) or the length of time between the two stages (p = 0.629; Table 1).

**Table 1:** Age at diagnosis, age at surgery, referral time and waitlist time in the studied patients IQR: interquartile range; *significant at *p* < 0.05.

		Groups			p-value
		Total (n = 175)	≤18 months (n = 66)	>18 months (n = 109)	
Age at diagnosis (months)	Range	<1-156	<1-15	<1-156	<0.001*
	Median (IQR)	12 (3-24)	3 (2-7)	23 (17-33)	
Age at surgery (months)	Range	<1-156	<1-18	19-156	<0.001*
	Median (IQR)	24 (13-36)	12 (11-15)	33 (24-48)	
Duration between diagnosis at pediatric surgery clinic and surgery (months)	Range	<1-72.0	<1-17	<1-72	0.558
	Median (IQR)	8 (3-12)	8 (5-11)	7 (3-16)	
Age at stage I (months)	Range	12-88	12-18	24-88	0.057
	Median (IQR)	18 (14-25)	16 (13-18)	25 (24-88)	
Age at stage II (months)	Range	24-102	24-27	30-102	0.057
	Median (IQR)	27 (24-48)	25 (24-27)	48 (30-102)	
Duration between stage I and stage II (months)	Range	6-23	7-13	6-23	0.629
	Median (IQR)	12 (7-14)	10 (8-13)	14 (6-23)	

**Figure 2:**
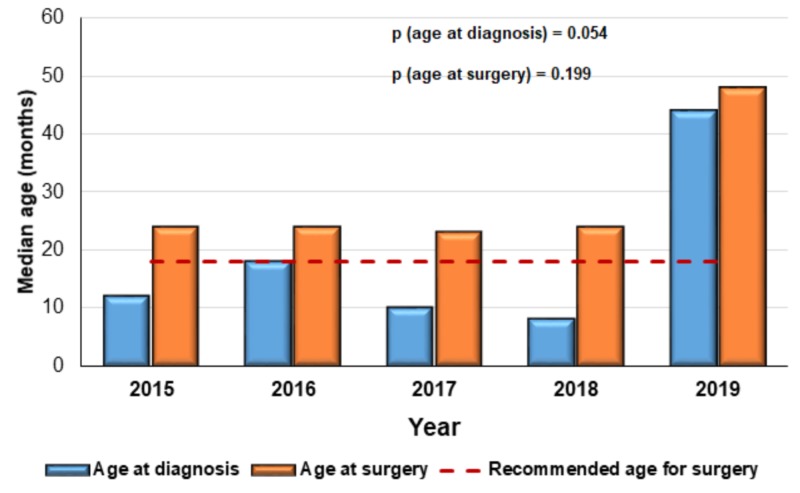
Median age at diagnosis and at surgery in the studied years. The dashed red line represents 18 months of age, which is the highest age limit according to clinical guidelines

The median age of patients at diagnosis and at surgery was compared among the years studied, revealing no significant difference.The highest median age at diagnosis and at surgery were in 2019. The median age at surgery was substantially higher than the recommended age (before or at 18 months old; Figure 2).

Patients were divided into two groups according to their age at surgery. Only 66 cases out of 175 (37.7%) underwent surgery during the recommended age period (at/before 18 months old). The median age at diagnosis was significantly higher in group 2 than in group 1 (23 months versus three months, *p* < 0.001). However, there was no significant difference between the two groups with respect to the duration between diagnosis and surgery (*p* = 0.558), age at stage I (*p* = 0.057), age at stage II (*p* = 0.057) or the length of time between the two stages (*p* = 0.629).

The most common site of UDT among the studied patients was inguinal (80.6%). The testis was not seen in 7.4% of cases. Most cases were unilateral, while only 24.6% of cases were bilateral. In most cases, the UDTs were palpable (73.1%), while only 25.7% of cases were impalpable. There was no significant difference between the studied groups with respect to the site or side of UDT (*p* > 0.05). Significantly higher percentages of patients with early orchidopexies had unseen (13.6% versus 3.7%, *p* = 0.019) and impalpable (37.9% versus 18.3%, *p* = 0.001) UDTs than did patients with delayed operations (Table 2).

**Table 2:** Year of surgery and clinical findings in the studied patients *Significant at *p* < 0.05

		Groups						p-value
		Total (n = 175)		≤ 18 months (n = 66)		> 18 months (n = 109)		
Site of UDT	Intra-abdominal	10	5.7%	4	6.1%	6	5.5%	1.000
	Inguinal	141	80.6%	49	74.2%	92	84.4%	0.100
	Upper scrotum	12	6.9%	5	7.6%	7	6.4%	0.766
	Ectopic	2	1.1%	0	0.0%	2	1.8%	0.527
	Not seen	13	7.4%	9	13.6%	4	3.7%	0.019*
Side of testis	Bilateral	43	24.6%	13	19.7%	30	27.5%	0.401
	Unilateral, left	75	42.9%	32	48.5%	43	39.4%	
	Unilateral, right	57	32.6%	21	31.8%	36	33.0%	
In clinical examination, testis was	Impalpable	45	25.7%	25	37.9%	20	18.3%	0.001*
	Palpable	128	73.1%	39	59.1%	89	81.7%	
	One palpable and the other impalpable	2	1.1%	2	3.0%	0	0.0%	

Approximately one-quarter of cases had an associated operation at the same time as the orchidopexy, the most common of which was circumcision, followed by herniotomy in 16 cases (9.1%) and hydrocelectomy in three cases (1.7%).

With respect to the surgical approach used, the majority of cases were operated upon using then open technique (85.7%). The open technique was used in all palpable UDTs, while the laparoscopic technique was used for impalpable testes. The size of the UDT was average in nearly half the cases, small in 44.6% and atrophic/vanishing in 6.4% of cases. No significant associations were found between the time of surgery and the surgical approach, the size of UDTs or associated operations (Table 3).

**Table 3:** Type and findings of surgery in the studied patients N/R, not recorded

		Groups						p-value
		Total (n = 175)		≤ 18 months (n = 66)		> 18 months (n = 109)		
Surgery	Open	150	85.7%	56	84.8%	94	86.2%	0.826
	Laparoscopic stage I	22	12.6%	8	12.1%	14	12.8%	0.889
	Laparoscopic stage II	7	4.0%	5	7.6%	2	1.8%	0.105
Intraoperative findings	Average size	86	49.1%	32	48.5%	54	49.5%	0.875
	Small size	78	44.6%	30	45.5%	48	44.0%	0.869
	Vanishing atrophic	11	6.4%	3	4.6%	8	7.5%	0.538
	N/R	3	1.7%	1	1.5%	2	1.8%	
Other associated operation at the same time		46	26.3%	21	31.8%	25	22.9%	0.196
Type of associated operation at the same time	Circumcision	27	15.4%	14	21.2%	13	11.9%	0.099
	Herniotomy	16	9.1%	5	7.6%	11	10.1%	0.576
	Hydrocelectomy	3	1.7%	1	1.5%	2	1.8%	1.000
	Meatotomy	2	1.1%	1	1.5%	1	0.9%	1.000
	Orchidectomy	1	0.6%	1	1.5%	0	0.0%	0.377
	Subcoronal hypospadias repair	1	0.6%	0	0.0%	1	0.9%	1.000
	Frenotomy	1	0.6%	0	0.0%	1	0.9%	1.000

The surgeries were eventless in the majority of cases, as complications were reported only in 4.0%. Wound infection was the most commonly recorded complication (three cases, 1.7%). No significant associations were found between the time of surgery and the occurrence or type of complications (Table 4).

 

**Table 4:** Postoperative complications in the studied patients N/R, not recorded

		Groups						p-value
		Total (n = 175)		≤ 18 months (n = 66)		> 18 months (n = 109)		
Complications	Absent	166	94.9%	65	98.5%	101	92.7%	0.254
	Present	7	4.0%	1	1.5%	6	5.5%	
	N/R	2	1.1%	0	0.0%	2	1.8%	
Reported complications	Wound infection	3	1.7%	1	1.5%	2	1.9%	1.000
	Inguinal hernia	2	1.2%	0	0.0%	2	1.9%	0.525
	Hydrocele	1	0.6%	0	0.0%	1	0.9%	1.000
	Testicular retraction	1	0.6%	0	0.0%	1	0.9%	1.000

Cox regression analysis was conducted to identify potential risk factors for delayed orchidopexy. The included variables in the analysis were age at diagnosis, site and side of the UDT and palpability of the testis on clinical examination. We found that age at diagnosis was the only significant risk factor that affected the time of surgery (HR = 1.015, *p* <0.001; Table 5).

**Table 5:** Cox regression to assess risk factors for delayed orchidopexy HR: hazard ratio; CI: confidence interval; *significant at *p* <0.0

		Wald	p-value	HR	95.0% CI for HR	
					Lower	Upper
Age at diagnosis (months)		23.647	<0.001*	1.015	1.009	1.021
Site of UDT	Intra-abdominal	0.003	0.954	0.949	0.162	5.579
	Inguinal	0.000	0.984	1.019	0.163	6.356
	upper scrotum	0.007	0.932	0.918	0.127	6.638
	Ectopic	1.370	0.242	2.692	0.513	14.135
	Not seen	0.991	0.320	0.336	0.039	2.880
Side of testis (bilateral)		1.257	0.262	1.297	0.823	2.043
Impalpable UDT		0.239	0.625	0.843	0.424	1.674

## Discussion

The present study included 175 patients who underwent orchidopexy from January 1, 2015 to March 30, 2019, with an average number of 40 cases operated annually. Similar rates were recorded from Saudi Arabia. An audit by Shareef et al. at King Fahad Hospital, Al Baha reported that 116 patients underwent orchidopexy over the course of three years, with an average of 39 cases per year [7].

Higher and lower rates of orchidopexies were reported by other studies. An audit conducted by Alsaywid in Sydney, Australia reported that 169 orchidopexies were performed within a period of two years [8]. Much lower rates were reported by other studies, such as the one conducted in Pakistan reporting 159 cases in 10 years [9]. 

The orchidopexy operation rate at our institution showed an unexplained decrease in 2018 (8.4%) compared to the previous years (12.7 - 14.7%). This decrease may be attributed to either a true decline in the incidence of UDT in Tabuk or a failure in the diagnosis and management of the disease. 

Studies from other countries have shown varying temporal trends of the annual rate of orchidopexy. Springer et al. studied orchidopexies performed in Austria between 1993 and 2009 and found the total rate of orchidopexy to rise continuously throughout the study period [10]. However, an Australian study demonstrated a true decline in the number of orchidopexies from 1999 to 2006 in Victoria [11]. The authors attributed this decline to changes in screening practices in the state of Victoria. We did not find similar studies that analyzed temporal trends in orchidopexies during our study period.

Surgery is a crucial treatment for UDT. Earlier surgery can prevent infertility and decrease the development of testicular cancer [4].

The recommended age of a patient at orchidopexy has changed considerably over the past several years. The guidelines issued by the American Urological Association, the American Academy of Pediatrics and the European Association of Urology/European Society for Pediatric Urology recommended that treatment should be started at the age of six months and be completed by the age of 12 months or, at the latest, 18 months [5,12-13].

In the present study, the median age at surgery was 24 months, with only 37.7% of cases undergoing orchidopexy during the recommended age period. Interestingly, this deviation from the recommendations of international guidelines persisted throughout the study, as a high median age at surgery was detected each year. The median duration between diagnosis and surgery was also relatively long (eight months).

Several studies from around the world have reported that the average age at orchidopexy was, unfortunately, higher than the age recommended by the guidelines. However, the reported ages at diagnosis and at surgery-as well as the wait time for the operation-varied widely across these studies. 

Locally, Neel studied orchidopexies in two hospitals in Riyadh, Saudi Arabia, and found that 45% of patients were diagnosed with UDT after one year of age [14]. Sharif et al. analyzed cases at King Fahad Hospital, Al Baha, SA, from 2011 to 2013. They reported that the mean age at surgery was almost three years, with 41.3% of boys undergoing orchidopexy being older than two years old [7]. Alsowayan and his colleagues conducted a study in the King Fahad Hospital, Al Khobar, Eastern Province, SA. They demonstrated that the median ages at diagnosis and at surgery were 13.7 months and 25 months, respectively, with an average wait time for the operation of 4.8 months [15].

Internationally, Schneuer et al. conducted a study in New South Wales, Australia, and reported the median age of orchidopexy to be 16.6 months [1]. Another Australian study in Victoria reported a much older age at the time of surgery, as approximately 55% were at least five years old [16]. Mallikarjuna et al. studied 30 cases at Chigateri General Hospital and Bapuji Hospital, Davangere, India. Their results demonstrated that 44.4% of boys presented for treatment after the age of three years [17]. A recent study conducted in the USA found that approximately 70% of boys with UDT underwent orchidopexy at least six months later than the recommended age [18]. 

However, a study by Alsaywid in Sydney, Australia, reported more favorable results, as only one-quarter of operated patients were older than 2 years of age, with the median age at surgery being 11 months [8]. Favorable results were also reported by Bajaj and Upadhyay, who conducted a retrospective study at Starship Children’s Hospital, Auckland, New Zealand. Their results showed that the median period between the clinic visit and the operation was 2.95 months, and the children’s median age at the time of surgery was 12.63 months. Moreover, 66% of cases had surgery before 18 months of age [19].

In our series of patients, cases of UDT were mostly unilateral (75.4%), and only 24.6% were bilateral. The most common side in unilateral UDT was the left (42.9% versus 32.6% on the right side). This finding contradicted the results of Mallikarjuna et al. and Ashley et al. who reported the most common side to be the right. [17,20]

The most common site of UDT was inguinal in our patients, followed by suprascrotal and intra-abdominal, which is consistent with the results found by Alsaywid and Mallikarjuna et al. [13,17].

In the present study, the UDT was impalpable in approximately one-quarter of the cases. This finding is in accordance with the findings of another study, which found nearly one-third of cases to have impalpable testes [8]. In another study, however, a much lower percentage of UDTs (10%) were impalpable [7]. 

Testes can be impalpable due to their site (intra-abdominal, intracanalicular, ectopic), size (atrophic) or congenital dysgenesis or agenesis. 

Most cases in this study (85.7%) were operated using the open surgical technique, while the laparoscopic technique was used only in 14.3% of cases. Sharif et al. reported the use of laparoscopy in only 9.65% of their cases [7]. The open approach is usually used for palpable testes, while laparoscopic surgery is usually reserved for impalpable UDTs [21].

We found that UDTs of average size constituted 49.1% of our cases, while 44.6% of UDTs were small and 6.4% were atrophic. The rate of atrophic UDTs reported in previous studies varied from 3.4% to 11.8% [7-8]. 

Complications were recorded in seven cases (4.0%) in our series. Similar rates of complications were reported by previous studies, ranging from 4.2% to 6.8% [7-8]. 

With respect to the causes of delayed orchidopexy for UDTs, we were not able to investigate all potential causes (such as educational level, socioeconomic status of the family, presence of medical insurance, presence of comorbidities and physicians’ experience) due to the retrospective nature of the study. However, it was evident from our results that the age at diagnosis affected significantly the time of surgery. Moreover, the wait time from diagnosis to surgery was relatively long. These factors were also detected by previous studies. Chen et al. in Taiwan showed that the age of patients at diagnosis, the number of clinic visits prior to surgery, the patient’s residence, the age of the physician making the initial diagnosis, and the age of the surgeon performing the surgery were significantly associated with delayed surgery [22]. Studies by Alsowayan et al. and Alhazmi et al. also suggested that delayed diagnosis and prolonged referral and wait times are important factors that contribute to delayed orchidopexy in Saudi Arabia [15,23].

Undescended testes can be diagnosed at birth in most cases. The diagnosis depends on the physician’s experience and the examination setup [24]. The delay in diagnosis and referral is attributed mainly to physicians’ deficient knowledge of UDTs and poor attitude. A survey in the United States found that only 30% of pediatricians and 14% of general practitioners recommended orchidopexy before the first year of life [25]. Another survey in the United States reported that 20% of the physicians did not refer children with UDT until puberty [26].

Pediatricians and family doctors have a duty to screen neonates for congenital anomalies, refer the cases to specialists and counsel the parents on the complications of delayed intervention. Consequently, there is a need to establish a protocol for newborn examination in which pediatricians screen for congenital anomalies and then refer patients to qualified, experienced pediatric surgeons. Physician training can improve their knowledge and attitude with respect to the timely diagnosis and referral of children with UDTs.

## Conclusions

In conclusion, in our study, the majority of cases underwent orchidopexy after the recommended age by international guidelines. The age at diagnosis seems to significantly affect the age at surgery. Educational programs that target general practitioners, pediatricians and parents are recommended to raise awareness of the importance of the early diagnosis and timed surgical management of undescended testes.
